# The SENSOR Study: Protocol for a Mixed-Methods Study of Self-Management Checks to Predict Exacerbations of Pseudomonas Aeruginosa in Patients with Long-Term Respiratory Conditions

**DOI:** 10.2196/resprot.6636

**Published:** 2017-05-19

**Authors:** Claire Roberts, Thomas L Jones, Samal Gunatilake, Will Storrar, Scott Elliott, Sharon Glaysher, Ben Green, Steven Rule, Carole Fogg, Ann Dewey, Kevin A Auton, Anoop J Chauhan

**Affiliations:** ^1^ Department of Research and Innovation Portsmouth Hospitals National Health Service Trust Portsmouth United Kingdom; ^2^ Department of Respiratory Medicine Portsmouth Hospitals National Health Service Trust Portsmouth United Kingdom; ^3^ School of Health Sciences and Social Work University of Portsmouth Portsmouth United Kingdom; ^4^ Aseptika Ltd Huntingdon United Kingdom

**Keywords:** COPD, bronchiectasis, pseudomonas, self-management

## Abstract

**Background:**

There are an estimated three million people in the United Kingdom with chronic obstructive pulmonary disease (COPD), and the incidence of bronchiectasis is estimated at around 0.1% but is more common in COPD and severe asthma. Both COPD and bronchiectasis are characterized by exacerbations in which bacteria play a central role. Pseudomonas aeruginosa is isolated from sputum samples from 4% to 15% of adults with COPD and is more likely to be isolated from patients with severe disease. Earlier detection of exacerbations may improve morbidity and mortality by expediting treatment. Aseptika Ltd has developed a system for patients to self-monitor important physiological measurements including levels of physical activity, peak flow, forced expiratory volume (FEV1), and biomarkers for P aeruginosa in sputum.

**Objective:**

We aim to test this system in 20 participants with *P aeruginosa* colonization and 10 controls with *Haemophilus influenzae*.

**Methods:**

We plan to recruit 30 adult participants with COPD or non-CF bronchiectasis who have cultured *P aeruginosa* or *H influenzae* during an exacerbation in the last 6 months. They must produce sputum on most days and should have been stable for 4 weeks prior to entry. Daily data collected will include symptoms, health care usage, medication, weight, FEV1, physical activity level, blood pressure, oxygen saturation, and temperature. Sputum and urine samples will be provided daily. These data will be analyzed to assess predictive value in detecting upcoming exacerbations. Qualitative data will be gathered through self-administered questionnaires and semistructured interviews to gather information on participant coping and their use of the technology involved.

**Results:**

Recruitment has been completed and results from the study should be available at the end of 2017.

**Conclusions:**

The SENSOR study aims to test a home-monitoring system in people with chronic airway infection and is currently underway.

## Introduction

### Overview

#### Burden of Respiratory Disease With Pseudomonas Aeruginosa

There are an estimated 3 million people in the United Kingdom with chronic obstructive pulmonary disease (COPD) [1]. Bacteria cause acute exacerbations that characterize the course of COPD, and these exacerbations are associated with substantial morbidity and mortality. In addition, bacteria are present in the lower airways of many adults with COPD, even during clinically stable periods, contributing to the airway inflammation that is a hallmark of COPD.

The observation that  *Pseudomonas aeruginosa* is isolated from sputum samples from 4% to 15% of adults with COPD in many cross-sectional studies [2-4] suggests that the bacterium is a relatively common cause of infection in this clinical context. *P aeruginosa* is more likely to be isolated from patients with severe disease [4], particularly among patients who require mechanical ventilation for severe exacerbations.

*P aeruginosa* may cause chronic infections in patients with COPD that are similar to those seen in patients with cystic fibrosis (CF). Strains of *P aeruginosa* that persist in the airways of adults with COPD demonstrate changes characteristic of chronic infection that are similar to changes observed in CF, supporting the conclusion that chronic  *P aeruginosa* infection occurs in the context of COPD.

The incidence of bronchiectasis in the United Kingdom is not certain. Chest x-ray reviews in the 1950s suggested an incidence of 100/100,000 with an increase in prevalence with age [5]. With the advent of advanced imaging techniques, up to 30% of patients with COPD and severe asthma are found to have features of bronchiectasis on computed tomography.

Within our center, exacerbations of COPD and bronchiectasis are managed through a combination of admission to a dedicated 76-bed unit and self-management by the patient/carer at home. Patients are provided educational literature, a care plan, and supplies of antibiotics and steroids for use at home. Patients are encouraged to recognize signs of exacerbation and to begin immediate treatment with antibiotics. They are requested to report when they have initiated antimicrobial therapy at home so this can be followed up within 2 days (but often do not report because they feel better). Best practice recommends that patients are followed up within 3 weeks after being admitted for exacerbation [1].

The Portsmouth Hospitals Trust (PHT) Respiratory Centre has created a culture of empowering patients through education and by transferring the skills required for self-management so as to reduce rates of admission [6]. The costs of providing skilled community staff for home visits has proved uneconomic but the cost burden of unscheduled admissions is equally high, necessitating innovative solutions to assist patients to self-manage.

Aseptika Ltd has developed a system for patients to self-monitor important physiological measurements including levels of physical activity, weight, peak flow, forced expiratory volume (FEV_1_) blood oxygenation, heart rate, wellness scores, clinical contacts diary, start of antibiotics and steroids, and 1 to 3 biomarkers of virulence of *P aeruginosa* in sputum. In a clinical trial sponsored by Papworth Hospital during 2013, data were collected by 15 CF participants and uploaded electronically to Activ8rlives, a Web-based data collection and viewing system developed by Aseptika. The study indicated that clinical parameters and sputum biomarker changes are likely to enable early detection of exacerbations in CF patients and have a role in highlighting treatment failure [7]. The Self-Management Checks to Predict Exacerbations of *Pseudomonas Aeruginosa* in Patients with Long-Term Respiratory Conditions (SENSOR) study aims to investigate if these tools can be more widely applied to other respiratory conditions such as COPD and non-CF bronchiectasis (NCFB). The Activ8rlives app operated from an iPad tablet uses wireless connectivity to home-use monitors to upload physiological and subjective scores and diary information directly to the Cloud-based Activ8rlives solution. The differing demographics between this study population and the Papworth population (ie, the older age of the Portsmouth population) will enable us to learn whether older patients with long-term conditions can be trained to use these monitoring devices.

#### Rationale for Study and Potential Impact

As described above, initial trials with Activ8rlives system with CF patients indicated a potential role of this technology in improving patient monitoring and self-management. This is particularly important in patients with chronic respiratory conditions for whom the staff-to-patient ratio is typically much lower than CF and for whom innovative solutions to better enable patients to monitor and self-manage their own condition are key to keeping their illness under control.

It is hoped that the results of this study will enable the self-care planning process that currently exists at PHT to be supported with the Activ8rlives technology to extend the effectiveness of our home hospital concept. Qualitative feedback from patients and carers will contribute to the continuing improvement and adaptation of the system.

The eventual addition of the Activ8rlives self-monitoring solution (including tests for sputum *P aeruginosa* virulence) as an assist for the patients and clinical staff and the provision of an information technology (IT) infrastructure for staff, which could simultaneously monitor and mentor many thousands of patients with respiratory conditions in the Portsmouth area without the costs incurred through undertaking face-to-face home visits, would have a significant impact both on patient health and National Health Service (NHS) costs. The development of this infrastructure and subsequent assessment of the effectiveness and cost effectiveness of the system would be assessed in a further large-scale randomized controlled trial.

### Aims and Objectives

#### Primary Objective

- To use longitudinal *P aeruginosa* sputum biomarker, telemetry, and symptom data to develop individualizable models to predict a *P aeruginosa* exacerbation in chronic non-CF respiratory disease

#### Secondary Objectives

- To investigate the correlation between *P aeruginosa* biomarkers and telemetry with clinical outcomes during and after treatment of an exacerbation with antibiotics
- To describe rates of adherence to data input by participants/carers
- To explore whether the self-management platform provided for data upload is feasible and acceptable for daily use in this clinical population
- To pilot questionnaires to collect data on health care use in this population

#### Exploratory Objectives

- To collect urine samples for future studies that may explore whether biomarkers present in urine can also contribute additional information to the exacerbation prediction model
- To perform molecular analysis for the *Haemophilus influenzae* group to corroborate microbiological tests in control groups.

## Methods

### Overview

In a mixed methods study, a longitudinal cohort of participants and their carers (where appropriate) will be asked to collect physiological, biological, and disease outcome data over a 6-month period. Neither participants nor the clinicians responsible for participant care will have access to the longitudinal data, and this information will not be used to make clinical decisions. The laboratory samples will be analyzed in a blinded manner. These data will then be analyzed to develop a model for predicting onset of exacerbations that can be built into a self-monitoring system.

Qualitative methods will be used to explore participant and carer experiences of using the technology and performing daily self-monitoring assessments. The expected duration of participation in the cohort will be 6 months, with an invitation to complete a self-administered questionnaire at baseline to inform a face-to-face semistructured interview with patients with or without carer joint participation at the end of the follow-up period.

### Primary and Secondary Endpoints and Outcome Measures

#### Primary Endpoint

An exacerbation will be defined as the initiation of antimicrobial therapy for respiratory symptoms either at home or on admission, with or without concomitant steroids or admission. For participants who are already on continuous antibiotics, an exacerbation will be defined as starting a course of different antibiotics or increasing the dose or frequency of existing antibiotics due to increased symptoms.

#### Secondary Endpoints

Treatment efficacy will be defined as the day at which antibiotic treatment for that episode is completed.

### Study Participants

#### Inclusion Criteria

The participant must meet all of the following criteria to be considered eligible for the study:

- Male or female, aged 18 years or above
- Diagnosed with at least one (or a combination) of COPD and non-CF bronchiectasis
- Two or more exacerbations with the same pathogen (*P aeruginosa* or *H influenzae*) proven on culture and treated with antibiotics within the last 12 months, one of which must have been within the last 6 months
- Exacerbation-free for the previous 4 weeks
- Producing at least 1 mL of sputum daily
- Must be capable of operating the self-monitoring devices and tablet-based IT system or have a carer capable of undertaking the measurements and collection, storage, and transport of samples
- Willing and able to give informed consent for participation in the study

#### Exclusion Criteria

The participant may not enter the study if any of the following apply:

- Suspected or confirmed diagnosis of CF
- Any condition likely to limit participant survival or adherence during the study period in the judgement of the clinician (eg, malignancy, cirrhosis of the liver)
- Currently taking part in any other research study

Carers will also be included in the qualitative interview and to aid participants with data upload. A carer in this study is defined as an adult (18 years or older) relative or friend who has frequent contact with the participant and will assist the participant to perform measurements or use the iPad tablet to upload data.

### Sampling and Sample Size

Using the sputum biomarkers to predict exacerbations, we based the sample size on the difference in exacerbation frequency in time periods when an exacerbation was predicted compared to time periods when no exacerbation was predicted. This will give an odds ratio of exacerbation following detection of sputum biomarkers.

Time periods will consist of approximately 10 days, of which it will be estimated that there may be on average 12 per participant (assuming an average of 4 months of follow-up). It is estimated that an exacerbation will occur in 60% of time periods in which it is predicted and 10% of time periods in which it is not predicted. It is also assumed that a predicted exacerbation will be made in only 1 in 12 time points (8% of all time points). Using a 5% significance level and 90% power, it is calculated a total of 120 time periods will be required.

However, as there are multiple time periods from the same participant, the data values are unlikely to be independent of each other and thus the sample size requires inflating. Assuming intraclass correlation of 0.18, a design effect of approximately 3 is calculated. This implies that 360 time periods are required for the analysis. This equates to a total of 30 participants for the study. The proposed 30 participants will consist of 20 participants with chronic *P aeruginosa* infection (10 with primarily COPD and 10 with NCFB) and 10 participants with *H influenzae* infection.

### Study Procedures

#### Recruitment

Potentially eligible patients, identified from clinical databases, will be contacted by a member of the research team and invited to attend an information event or specialist research recruitment clinic. The information event will be held locally on a Saturday or in the evening to make the event more convenient for patients and their carers. The event will allow patients to meet the research team, have an educational talk, and find out about the study. They will also be able to look at the devices and iPad app as well as ask any questions. Patients interested in participating in the study will be given a participant information sheet (PIS) and invited to come to a research clinic for screening and enrollment.

At the research clinic, patients will be reviewed by a clinical research fellow and screened for suitability for the study by the research team. Patients and carers will be able to view and try out the self-monitoring devices and tablet-based IT system. Capacity to operate these systems will be assessed as part of screening using a checklist, assessing the patient/carer confidence in using the devices and ability to perform the measurements. Those who meet eligibility criteria will be given a PIS and a carer information sheet if necessary. After adequate time to read, understand, and ask questions (at least 24 hours), patients will be invited back to the research clinic to give informed consent. Baseline data will be captured on paper case report forms (CRFs) and uploaded to a local study database, separate from the self-monitoring database held by Aseptika.

#### Participant and Carer Training

Volunteers will be trained by a research nurse and Aseptika personnel during a visit to the participant’s home. A home visit will clarify which 3G cellular network is available in their location (for Internet access) and will ensure that the participant has space to install a small dedicated freezer and that there is access for delivery.

Each participant will be provided with the following items and participant and carer will be trained on their use:

- Physical activity tracker: this will be set up for them and they will be shown how and when to wear it.
- Smart scale: the participant will be instructed how to weigh themselves and how this measures their body composition (they will be instructed to perform this in bare feet).
- Blood pressure cuff: the participant will be instructed how to take their own blood pressure and what the results mean.
- Pulse oximeter: the use of a pulse oximeter and how it works will be explained.
- Thermometer: the participant will be provided with a noncontact infrared thermometer and will be shown how to take their temperature.
- Peak flow meter: participants will be shown by the research nurse how to measure their peak flow and FEV_1_ using a simple, automated device.
- Sputum and urine samples: participant will be taught how and when to take a sample and how to store the sample in the containers provided in the freezer that will be supplied.
- iPad mini and questionnaires: participant will be trained on how to charge the iPad mini, how to switch it on and off, etc. A short questionnaire about symptoms, medications, and health care usage will be completed on the iPad mini using colorful self-explanatory series of screens within the Activ8rlives App. The display will be brightly colored, and any text (which will be kept to a minimum) will be large in size.

Participants will be able to use the iPad mini for personal use as well as for daily study use within the limits specified and agreed to in the participant consent form. Participants will be requested to take this equipment with them to the hospital if they are admitted and to continue recording these data in the hospital. Initial training will take approximately 60 minutes.

Following training, questions about use of the equipment will be answered by the research nurse and referred to the company in the event of technical difficulties with the IT system. The iPads will be supplied preconfigured with all software installed and an account for the volunteer already created and defined. A remote access technical support solution will also be preinstalled allowing the company to remotely access the iPad to resolve problems in the event of significant failures. If required, follow-up visits to the participants will be undertaken. Every effort will be made to ensure that the volunteer is adequately trained and supported for the duration of the study.

#### Study Assessments

**Figure 1:**
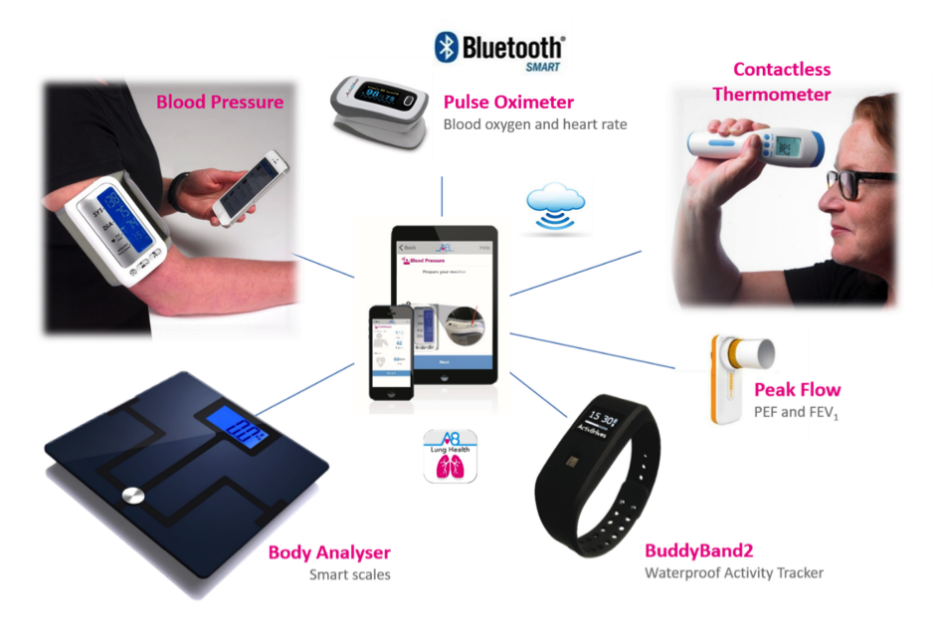
Equipment provided to participant to complete the assessments.

Participants will be asked to undertake the following once a day every day during a 6-month follow-up period, with assistance from carers as appropriate. Figure 1 illustrates the equipment that will be provided to the participant to facilitate these assessments.

Each participant will be provided with a 3G-enabled tablet (iPad) and will upload peak flow, FEV_1_, pulse rate, oxygen saturation, blood pressure, temperature, and physical activity data on a daily basis. The participants will be provided with a set of instructions about uploading the data onto the iPad. The participants will be blind to their continuous data over the duration of the study but will see the values generated daily on the screens of the devices. There will be no requirement for the participant to enter numerical values into the software as all monitoring devices connect directly reducing the risk of inaccurate data entry. The data collected on the iPad is automatically transmitted to the Cloud databases. No information is stored on the iPad and if the iPad is lost, there is no risk that these data could be accessed. The company is able to track the iPad’s location in the event that it is stolen or lost.

The information uploaded by the volunteers will be remotely reviewed daily by Aseptika’s Directors to ensure that data has been uploaded on a daily basis and there are no technical problems. Technical problems will be remedied remotely by the Aseptika technical staff. In the case of information not being uploaded, Aseptika’s investigator will initially inform the research nurse who will contact the volunteer by telephone to enquire if there have been any difficulties. Should any volunteers find the technology difficult to manage, they will be offered a further home visit for instructions and training.

#### Sample Production and Home Storage for Sputum and Urine Samples

- Containers will be prelabeled with the participant study number, expected contents (sputum or urine), and the day and date.
- Participants will be asked to fill the supplied pot with sputum and ensure the lid is correctly sealed.
- Participants will be asked to collect urine in the bulk container and secure the lid.
- They will then connect the Vacutainer to the cap of the urine container (as per supplied instructions), and the Vacutainer will begin to fill with urine.
- Bulk urine collection pot is to be discarded.
- Store both containers in sputum and urine collection bags inside of the supplied –20°C freezer.
- After 1 month, these will be transferred for bulk storage at the Queen Alexandra Hospital in a designated –70°C freezer and a new batch of containers will be provided.

#### Clinic Visits

Participants will be required to attend the clinic for a follow-up visit at months 3 and 6 (study completion) ± 2 weeks. Participants will attend their normal review with the medical team that usually involves lung function, sputum sample for culture, and clinical review. They will then have a research visit which will involve disease-specific control and quality of life questionnaires (Quality of Life–Bronchiectasis, Short Form Health Survey, St. George's Respiratory Questionnaire, EuroQol 5-Dimension Questionnaire) as well as noting any changes to treatment and exacerbations. The data collected from these visits will be recorded on paper CRFs which will then be uploaded by a member of the research team to a local study database with restricted access.

#### Health Economics

In preparation for a future cost-effectiveness trial of the self-management system, a questionnaire to collect health care use data will be piloted to capture resource use. These data will be analyzed descriptively. The daily questionnaire within the Activ8rlives app asks if participants have been in contact with a health professional or been in hospital in the last 24 hours. If they answer yes, they will be prompted to give more information about who they saw and for what reason. This will capture data on the participants’ health care use for their respiratory condition as well as other conditions.

#### Qualitative Methods

Qualitative methodology will be used to explore the psychosocial questions: How do the participant and main carer experience self-managing their condition under routine care? What is the participant’s expectation of taking part in the study? How does the participant experience collecting daily self-monitoring data and using the devices provided? Qualitative research seeks to describe, understand, and explain a particular phenomenon to make explicit the experiences and perceptions of the research subjects [8,9]. This is achieved by exploring the data (usually words) for conceptual definitions on how people perceive situations to provide explanations of why something happens in a particular way as well as looking for typologies or classifications of grouping of people (or situations) that tend to have common characteristics, opinions, and experience. Qualitative data will be collected at the end of the study (6 months) through face-to-face semistructured interviews with participants or paired interviews with carers, based on participant preference. Carers will not be interviewed separately.

All interviews will be guided by a semistructured topic guide although free discussion of experiences and ideas will also be encouraged. The semistructured interviews will be audiotaped and field notes taken to describe context, interview process, and initial theme development. A choice of venue, either at home or suitable hospital room, will be used. All qualitative interviews will be conducted by the same trained research fellow supported and supervised by another experienced qualitative researcher.

It is anticipated that the semistructured face-to-face interviews will take between 45 minutes to 1 hour to complete, depending on what the participant wishes to share. The interview will be terminated at any point the participant wishes to stop and this will not influence their subsequent treatment. As a small token of appreciation for time given to take part in the interviews, each participant will be offered a £10 (US $13) gift voucher on completion.

The interviews will be digitally recorded, transcribed verbatim, and entered into NVivo 10 (QSR International), a qualitative software package for systematic and transparent data management. All participant names will be removed from the transcripts to retain confidentiality. Care will be taken to always ensure any direct quotes used in study reports or papers to illustrate the findings will not be directly attributable to individuals.

#### Self-Administered Questionnaire

After consent and on entry to the main study, all patients but not carers will be asked to complete a self-administered questionnaire (to be developed with advice from the Patient Public Involvement [PPI] team members) with open and closed questions regarding self-awareness/perception of burden of disease, self-management including use of medication, identifying signs and symptoms of deterioration, problem-solving, seeking support/health care professional input during usual care as well as rationale and expectation of participation in the study and perceived barriers and enablers to successful completion in the study. We anticipate that this short self-administered questionnaire will take approximately 30 minutes to complete. The purpose of this self-administered questionnaire is to use individual responses to act as an “aide memoire” at the follow-up interviews.

#### Analysis

Data analysis will use the framework approach which provides a systematic, auditable, and rigorous analysis of qualitative data [10]. It is also more deductive than other thematic analysis approaches and ensures that focused data are collected to answer the clear research objectives of the study. Experienced facilitators will independently code all data. Scrutiny of the framework matrix will be sought to see if there is agreement with the categories generated. In addition, a member of the steering group not involved in data collection will be asked to independently read through a sample of the transcripts to generate a preliminary framework without seeing the original researchers’ list. In the case of disagreement, a solution will be sought to clarify the meaning of a code/theme developed until mutual consent is reached. The aim of this stage is to enhance the validity of the development of the conceptual framework and to guard against researcher bias. A narrative summary will be developed from the findings which includes comparison within case and across patient and carer’s perception and experience.

#### Discontinuation or Withdrawal of Participants From Study

Participants not complying with study requirements or failing to upload data or collect a sample will be retrained. Significant nonadherence (eg, noncollection of data and samples for a period of several weeks) may lead to withdrawal of the participant from the study by the study team. If participants and carers are not willing to continue data collection, they may decide to withdraw from the study and will be requested to return all the equipment.

#### Definition of End of Study

The end of study is the date of the last sample and data upload of the last participant follow-up date or the last qualitative interview, whichever comes last.

### Assessment of Safety

This is a noninterventional study and is therefore considered to be of no additional safety risk compared with usual clinical practice. All patients are expected to have a number of comorbidities and on-going symptoms due to their illness. All study procedures in use are the usual standard of care for this population and are not novel. The test used to observe the variation in levels of Exotoxin A is a new test but one that is validated and in itself provides no risk of harm to the participants; it is not performed on the participants themselves but on their sputum, which they would ordinarily be producing daily, and it will not change any clinical treatment as the results will not be available to the participant or their clinical team.

There is therefore no clear rationale for additional safety monitoring during the study period or for the expedited reporting of any serious adverse events. However, as study procedures are being conducted more frequently by the patients in their homes (as part of their daily routine), there may be an increased frequency of expected side effects or adverse events for some patients. The most likely adverse event during procedures is an increased risk of fainting for those participants who are susceptible to fainting during spirometry (specifically daily peak flow, which requires blowing rapidly and forcefully into a spirometer). Therefore, the following risk-adapted safety monitoring procedures are to be followed during the study period.

Participants will be asked to report any of the expected adverse events (dizziness or light-headedness and fainting) to the research team if they occur at a level which causes significant discomfort to the participant so that the research team can provide any further training or adjustments to the way in which the measurements are taken or reduce the frequency at which the participant is to take the measurement.

The chief investigator and study team will record and monitor any adverse events caused due to the increased frequency of the self-monitoring assessments. If there is any concern over these events or if they become unacceptable and in any circumstance cause the risk-to-benefit ratio to tip, they should be expedited to the sponsor and may be subsequently reviewed by the Research Quality Committee.

### Data Handling and Record Keeping

#### Data Collection Forms

The anonymous daily data generated by participants using the technology provided to them will be uploaded directly to the Web servers of Aseptika via 3G built into the iPad tablets provided to the participants. The anonymized data collected at the hospital visits at baseline, 3, and 6 months will be recorded on study-specific CRFs. The data recorded on these forms will then be uploaded by a member of the research team onto the local study database held at Portsmouth Hospitals Trust. This database will be password protected and kept on a secure NHS server with restricted access on a computer with restricted access in a locked room.

#### Data Management

Each volunteer will be given an account within Activ8rlives online system. Their name, NHS number, address, or any other identifying information will not be entered. Anonymity will be assured by giving each participant a unique study ID which will not be traceable to the participant. The unique study ID will be formed from 2 letters and 2 numbers (eg, SE01 for the first participant recruited into the study, SE02 for the second participant recruited).

Aseptika’s servers are located in the United Kingdom and have appropriate security measures. Access to these data will be restricted to Director-level personnel within Aseptika and to the research nurse for the purposes of technical support and to track progress of the study. Patients and carers, clinical staff, and laboratory staff will have no access to these data.

Data generated from sputum and urine analysis will be output to Excel (Microsoft Corp) spreadsheets and will be imported into the account for each volunteer to be correlated with other telemetry data. Other clinically relevant information (start of antimicrobial therapy, admission or other treatment) which is associated with an endpoint will be entered into the Activ8rlives system.

### Data Analysis

#### Description of Analysis Populations

The study is a nonrandomised cohort of a single group of patients. All subjects recruited into the study will be included in the data analysis.

#### Analysis of Endpoints

The first stage of the data analysis is to use data on the collected parameters (eg, peak flow, pulse rate, oxygen saturation, activity data) to predict when an exacerbation is likely. Control charts will be used to determine the boundaries of normal behavior for each parameter. Separate control charts will be used for each participant, as what constitutes normal behavior will vary from participant to participant. When a parameter strays from normal behavior (eg, exceeds 99% control limits), an exacerbation will be predicted.

To examine the association between predicted exacerbation and actual exacerbation, participant follow-up will be divided into periods of time (eg, 7-10 days). A comparison of actual exacerbation when exacerbation has and has not been predicted will be made. To allow for the repeat measurements over time from the same participants, the analysis will be performed using multilevel logistic regression. Additionally, the sensitivity and positive predictive value of the predictions will also be calculated. Estimated values will be presented along with corresponding confidence intervals. Further exploratory analysis using novel computing methods will be conducted.

Health care use data will be presented descriptively (eg, the frequency and type of health care contacts over the study period for different participant groups).

#### Procedure for Dealing with Missing, Unused, and Spurious Data

The primary analysis will be restricted to collected data only, without any data imputation. The distributions of the parameters collected will be assessed, and implausible values may be excluded from the analysis. Any data exclusions will be justified both clinically and statistically.

#### Procedures for Reporting any Deviations From the Original Statistical Analysis

Any deviations to the statistical analysis plan will be carefully documented and justified.

### Ethics

#### Overview

The study will not be initiated before the protocol and all study-relevant material such as informed consent forms and participant and general practitioner information sheets have received approval or favorable opinion from the Research Ethics Committee (REC) and the respective NHS research and development department. Any changes to protocol or relevant study documents will be approved by the sponsor. Should an amendment be made that requires REC approval, defined by REC as a substantial amendment, the changes will not be instituted until the amendment has been reviewed and received approval or favorable opinion from the REC and research and development departments. A protocol amendment intended to eliminate an apparent immediate hazard to participants may be implemented immediately providing that the REC is notified as soon as possible and an approval is requested. Minor amendments, defined by REC as nonsubstantial amendments, may be implemented immediately and the REC will be informed.

#### Participant Confidentiality

Study staff will ensure that participant anonymity is maintained. The participants will be identified only by initials and participant ID number on the CRF and any electronic database. All documents will be stored securely and only accessible by study staff and authorized personnel. The study will comply with the Data Protection Act which requires data to be anonymized as soon as it is practical to do so.

#### Benefits and Burdens to Participants

This study requires a high engagement and compliance rate from the study participants. The additional daily measurements, monitoring, and uploading of the information onto the study's website for 6 months will be an additional time burden for the participants and may add significantly to their already onerous treatment burden. Clear instruction, in-depth training, and on-going technical support for all of the study equipment along with regular input from an experienced and committed research team will help to mitigate any burden.

Participants will be required to store the various study equipment including a small study freezer, iPad mini, and self-monitoring gadgets in their own homes which may be a burden for some participants, especially those living in smaller properties. Participants will be made aware of the equipment they will be provided with and information on how much space they will need to store the equipment prior to consent. The capability to store the study equipment will be evaluated as part of a participant eligibility assessment prior to giving out participant information.

A research nurse and Aseptika trainer will need to complete a one-off visit to participants’ homes to deliver, install, and train the participant in the various study technology. To minimize the burden of this visit, participants will only ever be contacted by the research team, not the company, and a visit time will be scheduled in advance at a time that is convenient to the participant.

On completion of the entire study, each participant and their carer will be shown the data and what has been learned from their participation in the study. Participant benefits include being compensated £1 (US $1.30) a day for successfully completing the measurements and collecting samples, retaining the iPad mini and freezer, being provided with 3G connectivity for 6 months following completion of the study, and retaining ownership of the monitoring devices. Participants will be encouraged to continue the self-monitoring process unblinded thereafter and will have full access to the data they generate. The study will be reviewed by the Research Quality Committee at regular intervals.

### Patient Public Involvement

The study design is similar to that of a previous study carried out with 15 CF patients in Cambridge. Adaptations of the content and methods of data collection and the final design of the questionnaire on the iPad will be informed by a group of PPI members convened for the purposes of this study. These members will be patients at PHT with similar respiratory conditions and will be invited to attend the group by their respective clinicians. PPI members have also given input on the study design and implementation issues and reviewed the PIS and informed consent form. Some key issues raised by the group are listed below, and these will be addressed in the preparation of study implementation:

- How big is the freezer (concern over space at home)?
- How often will samples be collected (concern about storage of samples and consumables and possible expenses to bring samples to the hospital, plus ensuring that any staff who collect samples from the house will have appropriate ID)?
- Ease of use of the Vacutainer urine collection system for participants with comorbidities such as arthritis
- Recommended preprinting of labels
- Some apprehension about using an iPad
- Whether the participants who take part will get to know the results of the study
- What happens if participants wish to go on holiday?

## Results

The SENSOR study is now in data analysis. It is anticipated results will be available by the end of 2017. The use of technology by study participants was successful with high upload figures and product reliability.

## Discussion

The SENSOR study will provide a prospective evaluation of the efficacy of this home self-monitoring system in people with chronic airway infection. The data collected will allow us to determine the key elements of the self-monitoring system for prediction of exacerbations in order to reduce the burden on monitoring on patients in future.

## Abbreviations

CF: cystic fibrosis
COPD: chronic obstructive pulmonary disease
CRF: case report form
FEV1: forced expiratory volume in one second
IT: information technology
NCFB: non-cystic fibrosis bronchiectasis
NHS: National Health Service
PHT: Portsmouth Hospitals Trust
PIS: participant information sheet
PPI: public patient involvement
REC: Research Ethics Committee
SENSOR study: Self-Management Checks to Predict Exacerbations of Pseudomonas Aeruginosa in Patients with Long-Term Respiratory Conditions

## Appendix

Multimedia Appendix 1 Original peer review report (Portsmouth Hospitals NHS Trust).
