# Supplementary material for: The SENSOR Study: Protocol for a Mixed-Methods Study of Self-Management Checks to Predict Exacerbations of Pseudomonas Aeruginosa in Patients with Long-Term Respiratory Conditions
Source: JMIR Res Protoc. 2017 May 19;6(5):e89. doi: 10.2196/resprot.6636 (PMC5457528; doi:10.2196/resprot.6636)
Supplement: Multimedia Appendix 1 [file resprot_v6i5e89_app1.pdf]

Project Ref No/ID:

Portsmouth Hospitals NHS Trust

Research Office
1st Floor, Gloucester House
Queen Alexandra Hospital
Cosham
Portsmouth
PO6 3LY

Tel: 023 9228 6236
Fax: 023 9228 6037
Email: research.office@porthosp.nhs.uk

DESIGN REVIEW FORM

Front sheet details are confidential

▪ Name of Reviewer:	**Dr Fraser Brims**
▪ Institution/Organisation:	**Lung Institute of Western Australia**
▪ Reviewer's Signature:	FBrims

Project Ref No/ID:

RESEARCH DESIGN REVIEW FORM

Thank you for agreeing to provide a Design Review of the enclosed research proposal on behalf of the Portsmouth Hospitals NHS Trust. Please complete your assessment on this form and qualify your views where possible. The R&D Office staff can be contacted at any time should you have questions or concerns about the peer review process. Please note that the R&D Office will arrange for a separate review of strategic relevance or 'Topic Area'. Your assessment will be passed unattributed to the research team.

On completion of your review **please sign the front sheet** and return this form to the R&D Office.

<table>
<tr><td colspan="2">R&D Office Use Only
Project Details:</td><td colspan="2">**Dates:**</td></tr>
<tr><td>Title:</td><td>**The SENSOR Study: A mixed-methods study of SElf-management checks to predict exacerbatioNs of Pseudomonas aeruginosa in patients with long-term reSpiratORy conditions.**</td><td>Sent to Reviewer On:</td><td></td></tr>
<tr><td>Local Ref No:</td><td></td><td>To be Returned By:</td><td></td></tr>
<tr><td colspan="2">**Principle Investigator Details:**</td><td colspan="2">**Enclosures:**</td></tr>
<tr><td>Name:</td><td></td><td>Protocol:</td><td></td></tr>
<tr><td rowspan="2">Institution:</td><td rowspan="2"></td><td>C.V. Details:</td><td></td></tr>
<tr><td>Other:</td><td></td></tr>
</table>

Project Ref No/ID:

RESEARCH DESIGN REVIEW FORM

Please provide your views on any aspect of the project proposal, commenting specifically on the numbered areas identified in the left-hand column. Please then provide a summary of your views in the table overleaf. Both assessments will be copied and passed, unattributed, to the lead researcher.

**1. Research Quality**	
Please give your opinion on the overall quality of the design.<br><br>**Prompts:**	
**Background and literature:** Is the current state of knowledge outlined, well structured, coherent and well -referenced?	Yes
**Question:** Is there a clear research question/ purpose, which leads on from the background and literature?	Yes
**Objectives:** Are the objectives clearly stated, appropriate and achievable?	Yes
**Design:** Is there a rationale for the approach? Are the methods chosen suitable and appropriate? Is the sample population described with reasons given for sample size? Will the sample size provide meaningful data once analysed? Are the research tools (such as questionnaires /interviews) well structured, informed, and suitable for analysis?	Yes. What is the likelihood of drop out from the study? Should this be incorporated – or will the numbers allow for this?
**Analysis:** Is there an effective analytical plan?	Covered briefly - A formal statistical analysis plan needs to be developed to better define the analysis plan.

2. Research Planning & Practice	
Please give your opinion on the overall research planning and practice.<br><br>**Prompts:**<br><br>**Project Management:** Is there evidence of a well-structured and achievable plan? Is the timetable realistic? Is the project manageable given the resources identified?	Yes
**The Investigative team:** Does the team have the appropriate multi-disciplinary and multi-professional mix to undertake the study? Are members of the team suitably qualified to carry out the stated methods and design? Would you advise further collaboration to ensure the study can be managed effectively?	Yes – broad, encompassing necessary skills, and a clear track record of success.

Please enter an assessment for each of the criteria listed below by ticking the appropriate box. The confidence level box is provided so that you can indicate your confidence in assessing the review report against the specified criterion (H=High, M=Medium, L=Low).

Criterion / Assessment	Supported	Supported with Specific changes *	Not Supported	Confidence Level H/M/L
1. Research Quality	Y			H
2. Research Planning & Practice	Y			H
3. Overall assessment	Y			H

*** Please document any specific changes required, overleaf**

Project Ref No/ID:

Any General Comments/Overall Assessment

Please clearly state any **specific changes** you would require to the protocol/research team

Suggest consider defining ‘worsening symptoms’ in the primary endpoint – (eg worsening SOB, cough, sputum production)

Inclusion critera – is it correct to use the term ‘infected with PA or HI’ – should this be ‘previous culture or colonised with ‘ ?

Is it OK to assume an equal exacerbation rate for those with PA and HI ?

Thank you for taking the time to complete this design review on behalf of Portsmouth Hospitals NHS Trust.

Please sign the front sheet and return with enclosures to:

The Research Office
1st Floor, Gloucester House
Queen Alexandra Hospital
Cosham, PO6 3LY
Tel: 023 9228 6236
Fax: 02392 296037
Email: research.office@porthosp.nhs.uk
